# Noise-robust phase-space deconvolution for light-field microscopy

**DOI:** 10.1117/1.JBO.27.7.076501

**Published:** 2022-07-26

**Authors:** Tianyi Zhu, Yuduo Guo, Yi Zhang, Zhi Lu, Xing Lin, Lu Fang, Jiamin Wu, Qionghai Dai

**Affiliations:** a Tsinghua University, Tsinghua-Berkeley Shenzhen Institute, Beijing, China; b Tsinghua University, Department of Automation, Beijing, China; c Tsinghua University, Department of Electronic Engineering, Beijing, China

**Keywords:** light-field reconstruction, phase-space, fluorescence imaging, three-dimensional imaging

## Abstract

**Significance:**

Light-field microscopy has achieved success in various applications of life sciences that require high-speed volumetric imaging. However, existing light-field reconstruction algorithms degrade severely in low-light conditions, and the deconvolution process is time-consuming.

**Aim:**

This study aims to develop a noise robustness phase-space deconvolution method with low computational costs.

**Approach:**

We reformulate the light-field phase-space deconvolution model into the Fourier domain with random-subset ordering and total-variation (TV) regularization. Additionally, we build a time-division-based multicolor light-field microscopy and conduct the three-dimensional (3D) imaging of the heart beating in zebrafish larva at over 95 Hz with a low light dose.

**Results:**

We demonstrate that this approach reduces computational resources, brings a tenfold speedup, and achieves a tenfold improvement for the noise robustness in terms of SSIM over the state-of-the-art approach.

**Conclusions:**

We proposed a phase-space deconvolution algorithm for 3D reconstructions in fluorescence imaging. Compared with the state-of-the-art method, we show significant improvement in both computational effectiveness and noise robustness; we further demonstrated practical application on zebrafish larva with low exposure and low light dose.

## 1 Introduction

Light-field microscopy (LFM) is an elegant method of capturing various biological dynamics in 3D at a high-speed frame rate. It reserves the volume information of organisms through single shot that simultaneously captures the spatial and angular information. The information can be used to recover the 3D volume of the sample through deconvolution algorithms, making LFM popular in various biological applications, especially for large-scale neural activities in 3D observation.^1^^–^^8^ Furthermore, some hardware improvement schemes based on LFM, such as scanning LFM (sLFM),^9^^,^^10^ mirror-enhanced sLFM,^11^ confocal LFM,^12^ etc., have also been proposed.

**Fig. 1:**
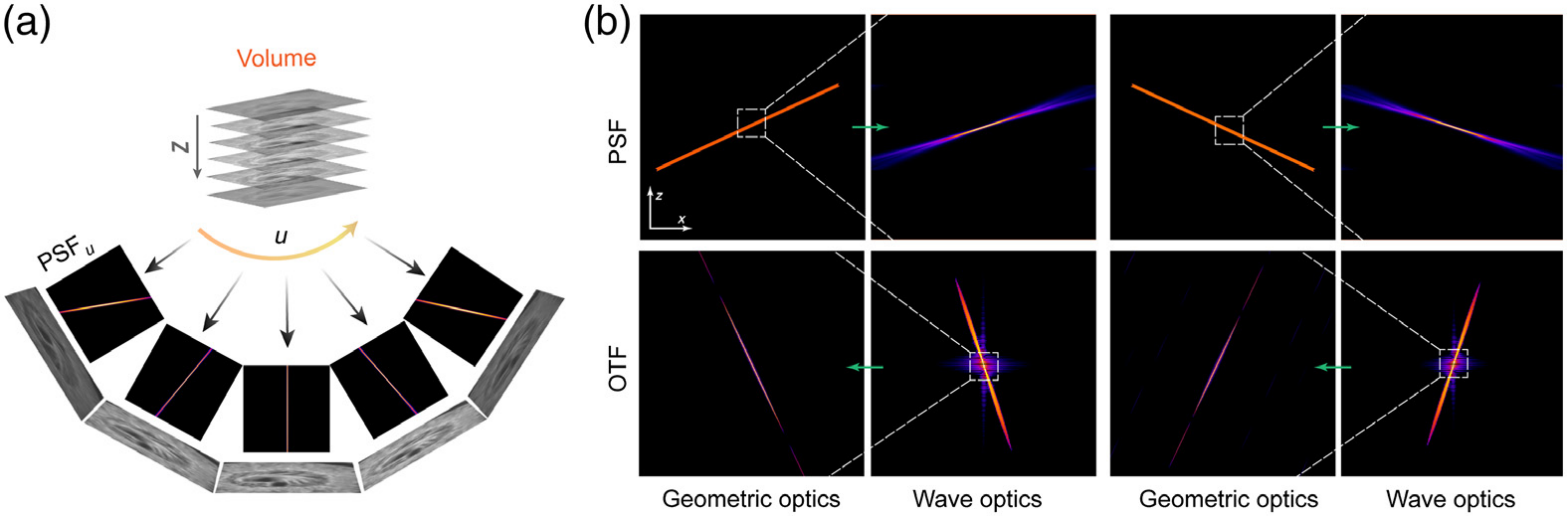
(a) Multiview images of LFM are the projections along with different phase-space PSFs based on wave optics, which can be leveraged to synthesize the high-resolution 3D volume in the Fourier domain, akin to the tomographic process. (b) The PSFs and optical transfer functions (OTF) of the LFM for different angular components are based on geometric optics and wave optics, respectively. The white box and the green arrow indicate the zoomed-in region of the PSF and the OTF. For samples with a scale much larger than the wavelength of the light, the PSF can be viewed as a straight line in the xz domain due to the large pixel size, which follows the common assumption of geometric optics. However, for samples with a scale comparable to the diffraction limit in microscopy, the diffraction effect will lead to an axially nonuniform PSF, which should be modeled by wave optics.

Many approaches for LFM deconvolution algorithms have been developed to facilitate the rapidly growing applications in recent years, such as the model-driven multiscale scattering model^13^ and Fourier-space model,^14^^,^^15^ data-driven dictionary learning model,^16^ and deep learning model.^17^^–^^19^ These LFM algorithms have gone through different stages. Geometric optics-based light field photography was first proposed to extend the realm of traditional photography with the ability of refocusing and 3D imaging.^2^^,^^20^ The Fourier slice theory then enhanced the efficiency of geometrical light field processing with orders of magnitude.^21^ However, the geometric model cannot describe the light field accurately in microscopy. Because the sampling size of complementary metal oxide (CMOS) is close to the diffraction limit, the geometric model results in severe resolution degradation in LFM. Broxton et al.^1^ proposed an LFM wave optics model to conduct the 3D deconvolution algorithm instead. They achieved a much higher spatial resolution for some axial planes. On the other hand, the wave optics model results in strong artifacts when approaching the native objective plane by frequency aliasing, which severely reduces the imaging contrast. Meanwhile, this model also causes high computational cost, limiting the scope of applications in LFM. Lu et al.^5^ proposed a phase-space deconvolution algorithm with a smooth prior in the phase-space domain to eliminate the artifacts and accelerate the algorithm by ten times. However, it still takes a very long time to reconstruct the sample volume. In addition, the noise robustness of these algorithms has not been analyzed accurately. LFM has a very strong background fluorescence from the out-of-focus planes, contributing a lot of shot noise to the phase-space measurements. Furthermore, a higher imaging frame rate leads to a much shorter exposure time and results in a low signal-to-noise ratio (SNR), which is an intrinsic limitation in fluorescent microscope.^1^ To solve these problems, we indicate that the multiview images in LFM are the projections along with different phase-space point spread functions (PSFs), which can be solved in a tomographic way (see Fig. 1 and Sec. 2). Fourier slice photography^21^ is represented as a simplified model when the object scale is much larger than the diffraction limit in our framework, which also shows the difference between geometric optics and wave optics on LFM in phase space [shown in Fig. 1(b)].

In this paper, we propose a new Fourier-phase-space LFM model with novel algorithms to solve the above-mentioned problems. The model leads to low computational cost and strong noise robustness in LFM 3D imaging. First, we transform our 3D deconvolution of LFM fluorescence samples to the Fourier domain by 3D Fourier transform. Then, we use random-subset ordering^22^ to accelerate the maximum likelihood estimation (MLE) of the phase-space model for LFM, and a 3D total-variation regularization is applied to utilize the sparse prior of the fluorescence samples. About tenfold improvement can be achieved in terms of the photon number required to achieve a similar SNR, compared with the previous method, with reduced computational costs.^5^ We evaluated the method by both numerical simulations and experiments on our customized multicolor LFM. High-speed heart-beating dynamics with 3D blood flows in a zebrafish larva were also observed at a volume rate of over 95 Hz. The limitations and potential future work are discussed in Sec. 4.

## 2 Method

### 2.1 Imaging Model

**Fig. 2:**
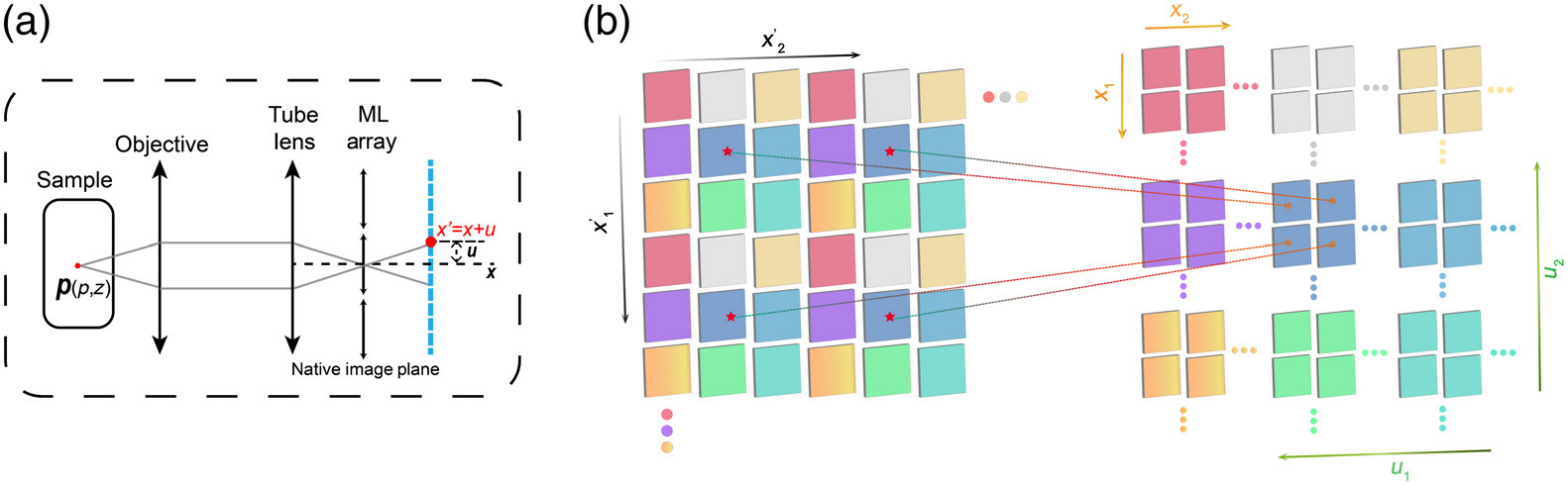
(a) Schematic of the imaging process by LFM with a microlens array inserted at the native image plane and a sensor placed at the back focal plane. P is a 3D point located at $\left(p,z)=(p_{1},p_{2},z\right)$. An arbitrary pixel on the sensor has a coordinate of $x=(x_{1},x_{2})$. The microlens in front of this sensor pixel has a center position of $x=(x_{1},x_{2})$, and the displacement of the sensor pixel to this center position x is $u=(u_{1},u_{2})$, corresponding to the angular coordinates of the phase-space measurements. (b) Illustration of the realignment procedure, which converts the raw light field measurement at the sensor plane into phase-space measurement with both the spatial coordinates $x=(x_{1},x_{2})$ and angular coordinates $u=(u_{1},u_{2})$.

We build our imaging model in the phase space based on wave optics. As shown in Fig. 2(a), we set the 3D point located at $\left(p,z)=(p_{1},p_{2},z\right)$. One pixel on the sensor with a coordinate of $x^{\prime }=(x_{1}^{\prime },x_{2}^{\prime })$ is realigned into phase space with a spatial coordinate of $x=(x_{1},x_{2})$, corresponding to the center position of the microlens in front of the sensor pixel, and an angular coordinate of $u=(u_{1},u_{2})$, corresponding to its relative position against the microlens center. To achieve angle projections from four-dimensional light-field capture, we introduce a realign-process that is described as Y(x,u)=L(x+u), where $L(x^{\prime })$ is the original light field image and $x^{\prime }=x+u$, which is also illustrated in Fig. 2(b). Therefore, we describe the phase-space PSF as^5^

$$h_{p}(x,p,z,u)=\int_{\omega_{x^{\prime }-x}}{\left\|K_{z}F_{\omega_{x^{\prime }-x}}\{U_{z}(x^{\prime }-p)\cdot t(x^{\prime }-x)\}\cdot s(u)\right\|}_{2}^{2}d\omega_{x^{\prime }-x},$$ (1)

where $K_{z}$ is a constant phase component related to z and $t(x^{\prime }-x)$ is a two-dimensional (2D) rectangle window function in spatial sampling, which is defined by the pitch size of a single microlens. $F_{\omega_{x^{\prime }-x}}(\cdot )$ represents the 2D Fourier transform based on the spatial frequency. $\omega_{x^{\prime }-x}\cdot s(u)=\mathrm{rect}((\omega_{x^{\prime }-x}-u)/d_{s})$ is also a 2D rectangle function in the spatial frequency domain with a window size determined by the sensor pixel size after each microlens ($d_{s}=6.5\;\mu m$ in our system). $U_{z}(x^{\prime }-p)$ is the analytical model for the complex field at the native image plane, generated by a point source $\left(p,z)=(p_{1},p_{2},z\right)$, which is described by Debye theory.^1^

So the imaging process is modeled in phase space as

$$Y(x,u)=\int_{z}\int_{p}g(p,z){\left|h_{p}(x,p,z,u)\right|}^{2}dp\;dz,$$ (2)

where g(p,z) is the intensity distribution of the 3D volume and Y(x,u) is the captured LF image after pixel realignment. As the phase-space PSF has been proved to be spatially-invariant for each angle, our imaging model is now simplified as

$$Y(x,u)=\int_{z}g(x,z)*H(x,z,u)dz,$$ (3)

where $H(x,z,u)={\left|h_{p}(x-p,z,u)\right|}^{2}$ and * represents the operator of 3D convolution. Because the background constant can be removed directly from the measurements and the shot noise is the main noise resources for fluorescence imaging, the final measurement is illustrated as

Y^(x,u)=Pois(Y(x,u)). (4)

#### 2.2 Fourier Space Representation

The MLE of this 3D convolution process is obtained through the 3D Richardson–Lucy (RL) algorithm.^1^ The general iteration formula for RL deconvolution is represented as

X(iter+1)=∑uωuBP(Y^u,Hu)∑uωu[BP(FP(X(iter),Hu),Hu)]·X(iter), (5)

where · represents the operator of matrix dot product; the division/represents matrix point division operator; FP(·) is the forward projection of the imaging model; $\mathrm{BP}(\cdot )={\mathrm{FP}}^{-1}(\cdot )$ is the backward projection process; $u=\{\Omega |(u_{1},u_{2})\in \Omega \}$ is the set of spatial frequency components, that is, the total number of the pixels after each microlens ($N_{u}=13\times 13=169$ in our system setup); and $\omega_{u}$ is the weight number that is corresponding to the energy distribution of the PSF H(x,z,u) as

$$w_{u}=\frac{\underset{z}{\sum }{\left\|H_{z,u}(x)\right\|}_{1}}{\underset{u}{\sum }\underset{z}{\sum }{\left\|H_{z,u}(x)\right\|}_{1}}.$$ (6)

The weight number $\omega_{u}$ is employed to compensate for the shot noise variances of different angles.

Then, the forward projection of the volume X is derived in Fourier space as

$$\mathrm{FP}(X^{\left(\mathrm{iter}\right)},H_{u})=\int_{z}X_{z}^{\left(\mathrm{iter}\right)}(x)*H_{z,u}(x)dz\;=F_{\left(x\right)}^{-1}\{F_{\left(x\right)}[(X^{\left(\mathrm{iter}\right)}(x,z)*H_{u}(x,-z)){|}_{z=0}]\}\;=F_{\left(x\right)}^{-1}\{F_{\left(x\right)}\{F_{\left(x,z\right)}^{-1}[(F_{\left(x,z\right)}(X^{\left(\mathrm{iter}\right)}(x,z))\cdot F_{\left(x,z\right)}(H_{u}(x,-z))){|}_{z=0}]\}\}\;=F_{\left(x\right)}^{-1}\{\int_{z}F_{\left(x,z\right)}(X^{\left(\mathrm{iter}\right)}(x,z))\cdot F_{\left(x,z\right)}(H_{u}(x,-z))dz\},$$ (7)

where F(·) represents the Fourier transform and $F^{-1}(\cdot )$ represents the inverse Fourier transform. To calculate the backward projection in Fourier space, we first define $A^{\left(z0\right)}(x)$ as a 3D volume that has the value of zero except for the central slice with the 2D value map A(x) and $H_{u}^{T(x)}(x,z)$ as the 3D PSF (on specific spatial frequency u) matrix transposed in 2D $x=(x_{1},x_{2})$. The replication function $R(A,N_{z})$ replicates the 2D matrix $A_{N_{x}\times N_{y}}$ into a 3D matrix $A_{N_{x}\times N_{y}\times N_{z}}^{\prime }$. Therefore, the backward projection of Eq. (7) is represented as

$$\mathrm{BP}(\mathrm{FP}(X^{\left(\mathrm{iter}\right)},H_{u}),H_{u})={\mathrm{FP}}_{X_{u}}^{\left(z0\right)}(x,z)*H_{u}^{T(x)}(x,z)\;=F_{\left(x,z\right)}^{-1}[F_{\left(x,z\right)}({\mathrm{FP}}_{X_{u}}^{\left(z0\right)}(x,z))\cdot F_{\left(x,z\right)}(H_{u}^{T(x)}(x,z))]\;=F_{\left(x,z\right)}^{-1}[R(F_{\left(x\right)}({\mathrm{FP}}_{X_{u}}(x)),N_{z})\cdot F_{\left(x,z\right)}(H_{u}^{T(x)}(x,z))],$$ (8)

where

$$F_{\left(x\right)}({\mathrm{FP}}_{X_{u}}(x))=\int_{z}F_{\left(x,z\right)}(X^{\left(\mathrm{iter}\right)}(x,z))\cdot F_{\left(x,z\right)}(H_{u}(x,-z))dz.$$ (9)

Notice that, as ${\mathrm{FP}}_{X_{u}}^{\left(z0\right)}(x,z)$ only has value at the central slice, its 3D Fourier transform is the replications of the 2D Fourier transform of its central slice. For the same reason, the backward projection of the light field data after realignment in phase space, which is also called phase-space measurements, is represented as

BP(Y^u,Hu)=Y^u(z0)(x,z)*HuT(x)(x,z)=F(x,z)−1[R(F(x)(Y^u(x)),Nz)·F(x,z)(HuT(x)(x,z))]. (10)

If Eqs. (8) and (10) are substituted back into Eq. (5), we finally get the iteration formula for the MLE of the phase-space model in the Fourier domain

X(iter+1)(x,z)=X(iter)(x,z)·∑uωuF(x,z)−1[R(F(x)(Y^u),Nz)·F(x,z)(HuT(x))]∑uωuF(x,z)−1[R(∫zF(x,z)(X(iter))·F(x,z)(Hu(x,−z))dz,Nz)·F(x,z)(HuT(x))]. (11)

For the MLE solution, we need to go through every angle within one iteration, which will reduce the reconstruction speed. Random subset ordering is a recently-proposed method in x-ray computed tomography to accelerate the reconstruction while maintaining the noise robustness. Because the phase-space model of LFM can also be viewed as a tomographic process as described, we implement the random subset ordering here to balance the benefit of noise reduction of the MLE solution and the speed strength of the single-angle iteration method.^5^ We mix the measurements of two random angles together as a new measurement before the iteration procedure to enhance SNR. The combinations of two random angles approach can therefore preserve fast convergence while gaining robustness against shot noise. In this case, similar to the method of our Fourier phase-space model, we further reduce the computational cost with the iteration formula shown as

X(iter,u′+1)=BP(Y^u′,Hu′)BP(FP(X(iter,u′),Hu′),Hu′)·X(iter,u′), (12)

where

Y^u′=Y^u1+Y^u2,H^u′=H^u1+H^u2. (13)

#### 2.3 Total Variation Regularization

Fluorescence samples are usually sparse compared with normal photography because only the structures of interests are labeled in each fluorescence channel. To make full use of such sparsity, we further add a 3D total-variation (TV) regularization during each iteration to improve the noise performance

$$X^{\left(\mathrm{iter}+1\right)}(x,z)=\mathrm{TV}(X^{\left(\mathrm{iter}\right)}(x,z)).$$ (14)

**Fig. 3:**
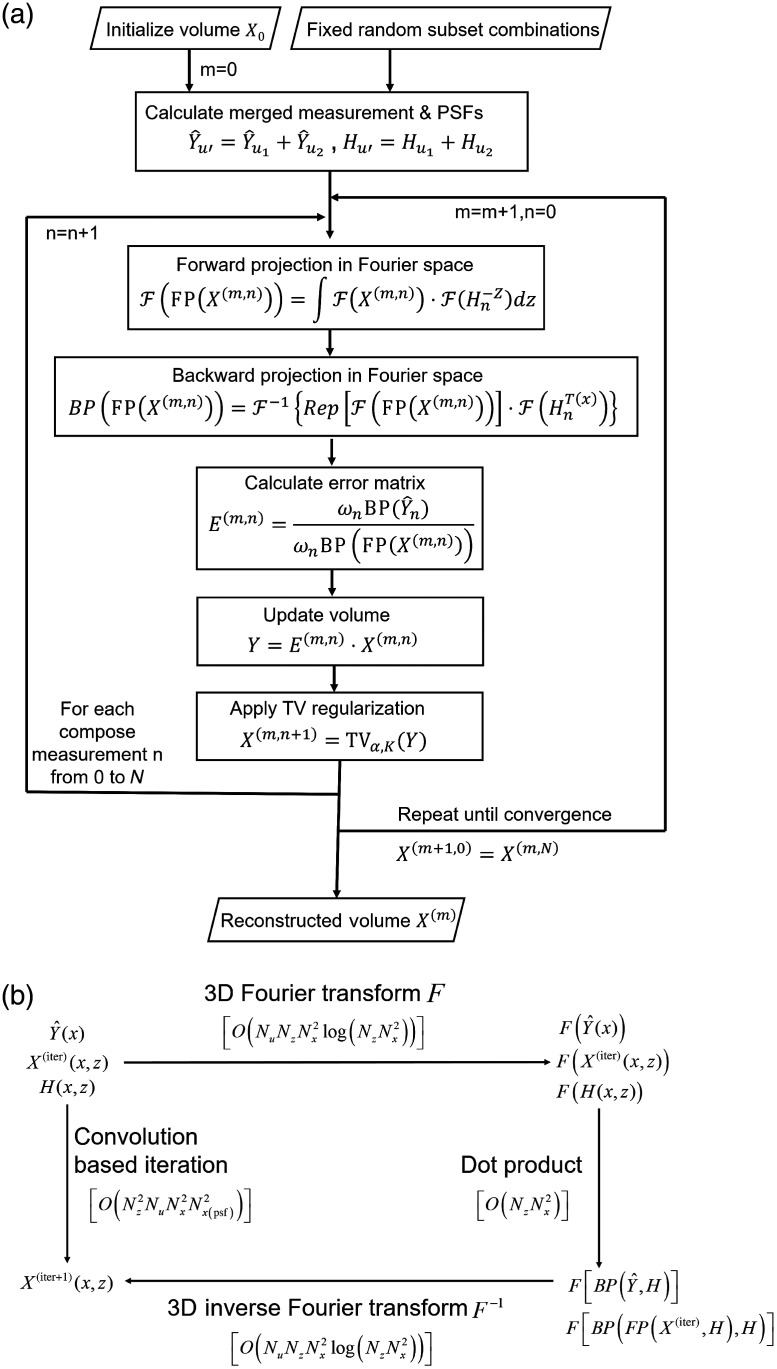
(a) The pseudocode of our Fourier phase-space deconvolution. (b) Comparison between Fourier phase-space deconvolution and traditional deconvolutions of LFM in the spatial domain with analysis of the time complexity for every major operation.

In practice, for each iteration, we update the volume by RL deconvolution first and then employ TV regularization by K times:^23^

$$X^{\left(\mathrm{iter},k\right)}=X^{\left(\mathrm{iter},k-1\right)}-\alpha \frac{v^{\left(k-1\right)}}{{\left\|v^{\left(k-1\right)}\right\|}_{l_{2}}},$$ (15)

where

$$v^{\left(k-1\right)}={\frac{d{\left\|X\right\|}_{\mathrm{TV}}}{dX}|}_{X=X^{\left(\mathrm{iter},k-1\right)}}\;{\left\|X\right\|}_{\mathrm{TV}}=\underset{x,z}{\sum }\sqrt{{\left(D_{x1}\right)}^{2}+{\left(D_{x2}\right)}^{2}+{\left(D_{z}\right)}^{2}},$$ (16)

where $D_{x1},D_{x2},\text{and }D_{z}$ correspond to the gradients along different dimensions. We chose $K=k_{\max}=1$ and α=3.0 for both the numerical simulations and experimental reconstructions. The whole process of our Fourier phase-space deconvolution is illustrated by the pseudocode shown in Fig. 3.

## 3 Results

To quantitatively analyze the effectiveness of the Fourier-domain phase-space model, we conducted numerical simulations on a denoised 3D data obtained by a commercial confocal microscope. For further evaluation of the improvement, we compare our results with the state-of-the-art algorithm (Lu et al.^5^), which has eliminated the reconstruction artifacts and obtained tenfold improvement in speed over traditional 3D deconvolution algorithms.^1^ The reduction of the computational cost of our method is analyzed in theory and a noise-free deconvolution simulation. Different shot noise levels are employed to show the noise robustness of our algorithm, especially for the TV regularization. The reconstruction quality is evaluated by the structural similarity (SSIM) index against the ground truth 3D data. Experimental results are conducted on a custom-built two-color LFM, with the reconstruction comparisons also shown in Video 1.

### 3.1 Simulation for Computational Cost

To fairly compare the time complexity of the phase-space deconvolution in the spatial domain and Fourier domain, we note that the PSF and the phase-space measurements may not share the same size. The size of PSF is usually smaller than that of phase-space measurements due to the memory limitation of the computer during calculation. Here, we use $N_{x}=(N_{x1},N_{x2})$ as the measurement size and $N_{x(\mathrm{PSF})}=(N_{x1(\mathrm{PSF})},N_{x2(\mathrm{PSF})})$ to define the size of PSF. For simplicity, we set $N_{x}=N_{x1}=N_{x2}$. As shown in Fig. 3(b), traditional algorithms^1^^,^^5^ used multiple 2D convolutions in the spatial domain, so the time complexity in the spatial domain is estimated as

$$T_{\mathrm{conv}}=O(N_{z})\times O(N_{u})\times O(N_{z}N_{x}^{2}N_{x(\mathrm{PSF})}^{2}+N_{x}^{2}N_{x(\mathrm{PSF})}^{2})\;=O(N_{z}^{2}N_{u}N_{x}^{2}N_{x(\mathrm{PSF})}^{2}).$$ (17)

As shown in Fig. 3(b), our Fourier phase-space deconvolution used 3D Fourier transform in both the forward and backward projections. By employing fast Fourier transform, the time complexity of our algorithm is estimated as (for simplicity, we have $N_{z}<N_{x}$)

$$T_{\text{fourier}}=O(N_{u})\times O(N_{z}N_{x}^{2}\;\log(N_{z}N_{x}^{2})+N_{z}N_{x}^{2})\;=O(N_{u}N_{z}N_{x}^{2}\;\log(N_{x})).$$ (18)

It should be noted that we resize the PSF as the same size as the phase-space measurements when doing the Fourier transform with zero padding, so the complexity should be computed based on the $N_{x}$. The ratio of time complexity between both algorithms is

$$\frac{T_{\mathrm{conv}}}{T_{\text{fourier}}}=O(\frac{N_{z}N_{x(\mathrm{psf})}^{2}}{\log(N_{x})}).$$ (19)

Such a ratio indicates the reduction of computational cost in the Fourier phase-space model, especially for a large volume with larger PSF size and more axial slices.

**Fig. 4:**
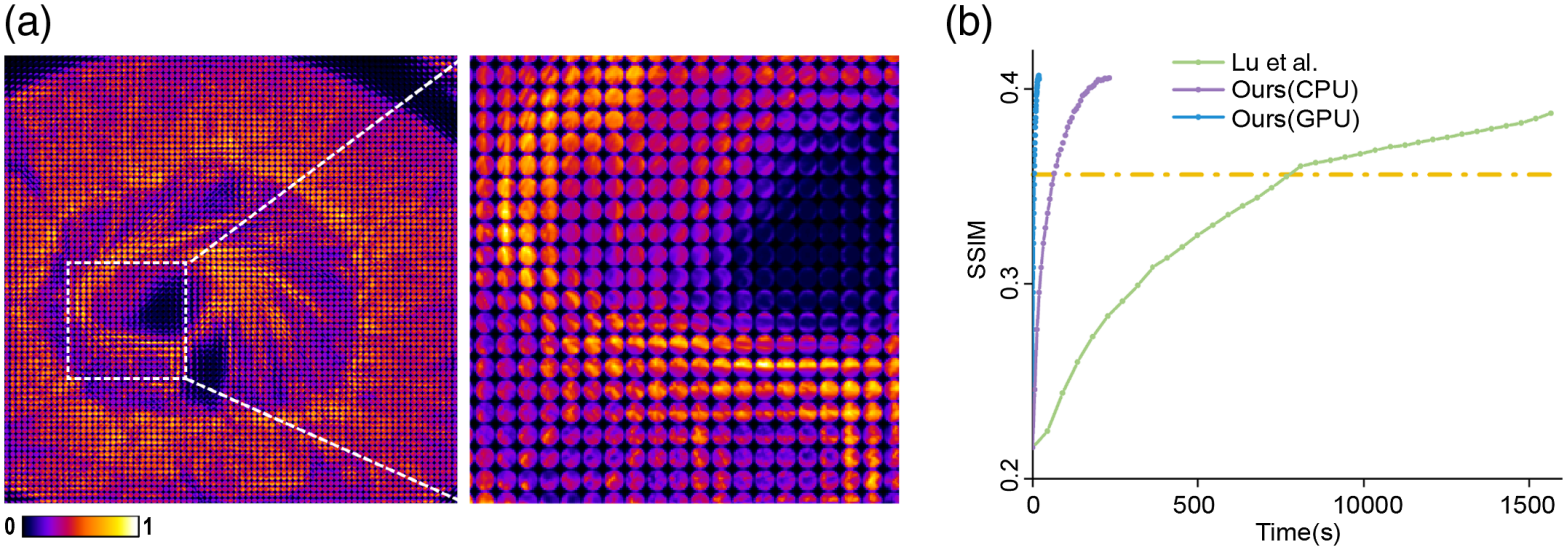
The computational efficiency of the proposed Fourier phase-space deconvolution. (a) The simulated light field measurements of a testis slice of a pig with a close-up are shown on the right. (b) The convergence curves of different algorithms. Under the same CPU computational platform, our approach significantly reduces the computational complexity and improves the convergence speed compared with the state-of-the-art algorithm, which can be further accelerated under the GPU platform. At the volumetric reconstruction SSIM around 0.35, our approach achieves two orders of magnitude reduction of the computational cost. Our approach requires only 50 and 655 s under GPU and CPU platforms, respectively, to achieve the reconstruction SSIM of 0.35, whereas the state-of-the-art method requires 7664 s.

To quantitatively analyze the noise performance and the computational cost of our algorithms in the Fourier domain, a numerical simulation was conducted. The 3D data used for simulation was a testis slice of pig with autofluorescence excited by the light of about 488 nm. We obtained the 3D information from a commercial confocal microscope (Olympus FV1000) with a 60×/1.4 NA oil-immersion objective and scanning pixel size 100 nm at an axial step of 530 nm. The whole 3D volume has a voxel number of 781×781×101 with PSF size 741×741×101. The whole imaging process of LFM is simulated based on wave optics, corresponding to our experimental setup with a microlens array inserted at the image plane and a sensor placed at the back focal plane of each microlens. The F-number of the microlens array is set to be 23 to match the 0.5 numerical aperture (NA) of the objective after a magnification factor of 23. Each microlens with a pitch size of 100  μm covers about 13×13  pixels. The synthesized light field imaging is shown in Fig. 4(a).

We conduct all of the comparison algorithms on the same computer with a i9-9980XE CPU and RAM of 128 GB. All of the codes are implemented on MATLAB R2019a. The GPU version using the gpuArray function in MATLAB was conducted by NVIDIA RTX Titan. We show the convergence curves of the ptychographic reconstruction algorithms with the maximum iteration number of 5. About a tenfold improvement in speed is obtained by our algorithm, compared with the state-of-the-art algorithm,^5^ for the CPU processing. Such an improvement mainly results from the reduction of the computational cost in the Fourier phase-space model. We show the speed results in Fig. 4(b), indicating that a similar reconstruction performance can be achieved with much less time by our algorithm, especially after acceleration by a GPU. Different from the convolution process in the spatial domain, most steps in our Fourier phase space convolution are the 3D Fourier transform, which can be easily implemented by cuFFT for GPU acceleration. In this case, the reconstruction time can be reduced below 100 s, which is about 100 times faster than the previous method.^5^ The maximum reconstruction SSIM is quite low due to the resolution loss of LFM, which is a tradeoff for the snapshot 3D imaging capability.

#### 3.2 Simulation for Noise Robustness

**Fig. 5:**
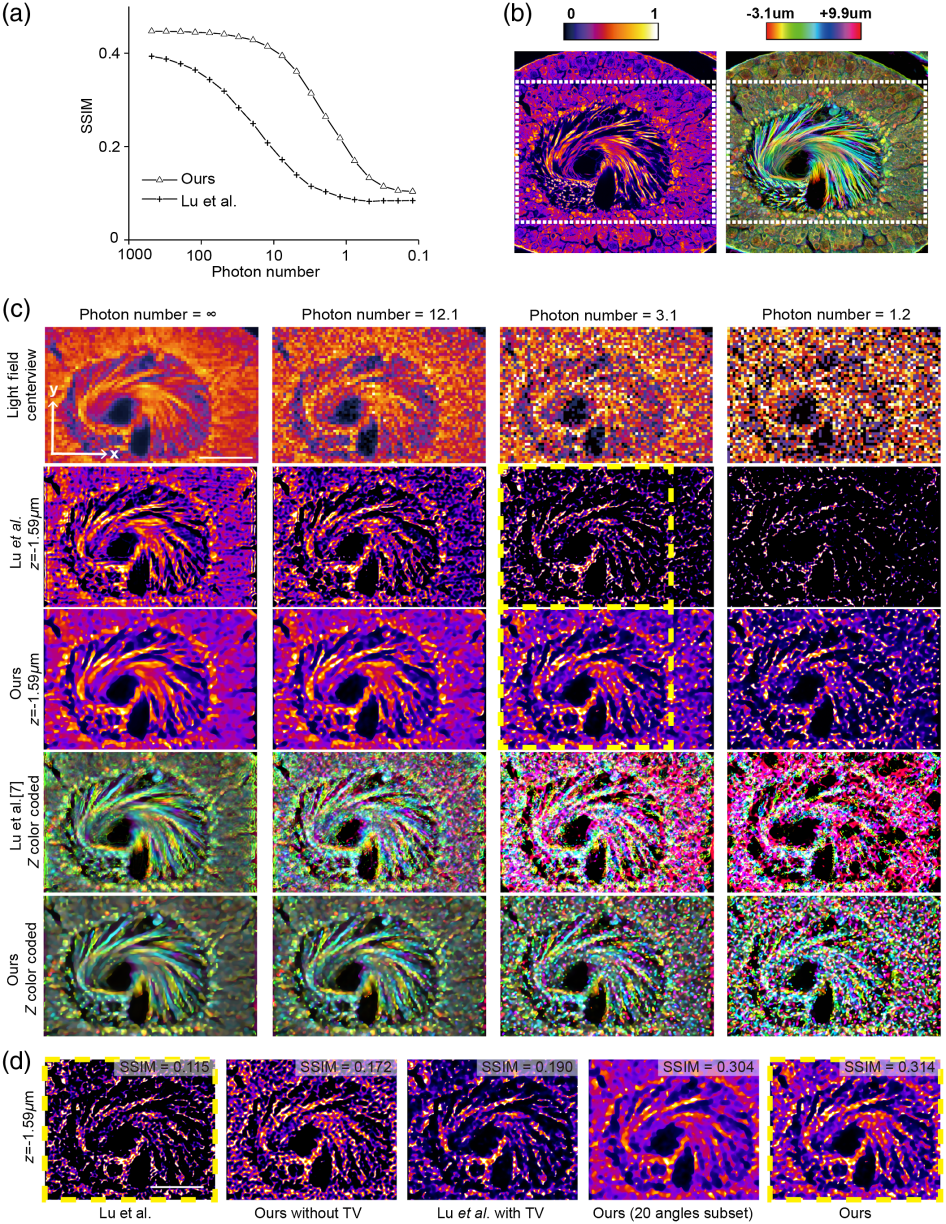
Noise analysis of our algorithms, compared with the state-of-the-art algorithm.^5^ (a) The SSIM of the reconstruction results by different algorithms at different shot noise levels, which is represented by the photon number per pixel of the light field measurements. (b) One axial slice of the ground truth is shown on the left, and the depth-coded projection of the ground truth is shown on the right with different colors corresponding to different axial planes. (c) Comparison of the proposed algorithm against the state-of-the-art algorithm^5^ with ptychographic iterations. Different columns correspond to different photon number counts per pixel labeled on the top. The infinity photon number means no noise is involved during the capturing process. The center views of the captured light field are shown in the first row. The axial slice and depth-coded projections reconstructed by different algorithms are shown below in different rows. (d) Ablation study on simulated experiment. Slice views and their volume SSIMs of five different reconstruction setups with the same shot noise level input (photon number = 3.1). The yellow boxes indicate the same regions in (c). Scale bar, 50  μm.

To address the noise robustness of our method, we applied different levels of shot noise to the synthesized light-field measurements and evaluated the reconstruction results by SSIM as shown in Fig. 5(a). The ground truth of a single slice and the depth-coded projection are shown in Fig. 5(b). For the depth-coded projection, we applied different hues for the voxels at different axial planes and averaged them along the axial dimension. The reconstructed results of the selected region in Fig. 5(b) under different noise levels including both single slices and depth-coded projections are shown in Fig. 5(c). We added different noise levels by the MATLAB function imnoise with parameter poisson and calculated its equivalent photon numbers per pixel in the simulation. The first row of Fig. 5(c) shows the center views of the synthesized data at different noise levels. All of the algorithms show strong robustness to noise, especially for the LF photon number below 100 per pixel. The shot noise in measurements will eventually introduce the speckle-like patterns over the reconstructed volume, which may cause fluctuations in the intensity. However, the performance of our results decreases more slowly than that of the previous algorithm^5^ in terms of SSIM.

From the color distribution in depth-coded projections, we can clearly observe the decrease of accuracy in depth with the increase of the noise. Such a degradation mainly results from the reduced contrast of the disparity between different angles, which is flooded by the shot noise. Our results show better performance, especially in the axial domain, because information from multiple angles is averaged together during backpropagation. TV regularization further provides a smoother distribution of the structures to avoid the speckles during synthesis. Even for the low photon number conditions (1 photon per pixel) shown in the fourth column of Fig. 5(c), our results with TV regularization can achieve similar performance as previous algorithm with a photon number of around 10 per pixel.

With the ablation study in Fig. 5(d), we show that both TV regularization and random subset can improve the phase-space reconstruction performance under low-light conditions, and our method benefits from both of these improvements. The subset combination merges its two measurements’ signals, which reduces shot noise, whereas the TV regularization reserves more effective information during each iteration. Therefore, the high-frequency component of every angular measurement is well reserved before they are led into the next angle subsets. Moreover, the 20 angles subset result with a decreased volume SSIM shows that the angle numbers are critical for both noise-robustness and convergence speed in our simulation because the MLE solution is equivalent to using all angles in every iteration but lacks convergence speed. Therefore, we choose the subset angle number of 2 as a trade-off for noise-robustness and convergence speed during the reconstruction process.

#### 3.3 Experiment on Zebrafish Larva

**Fig. 6:**
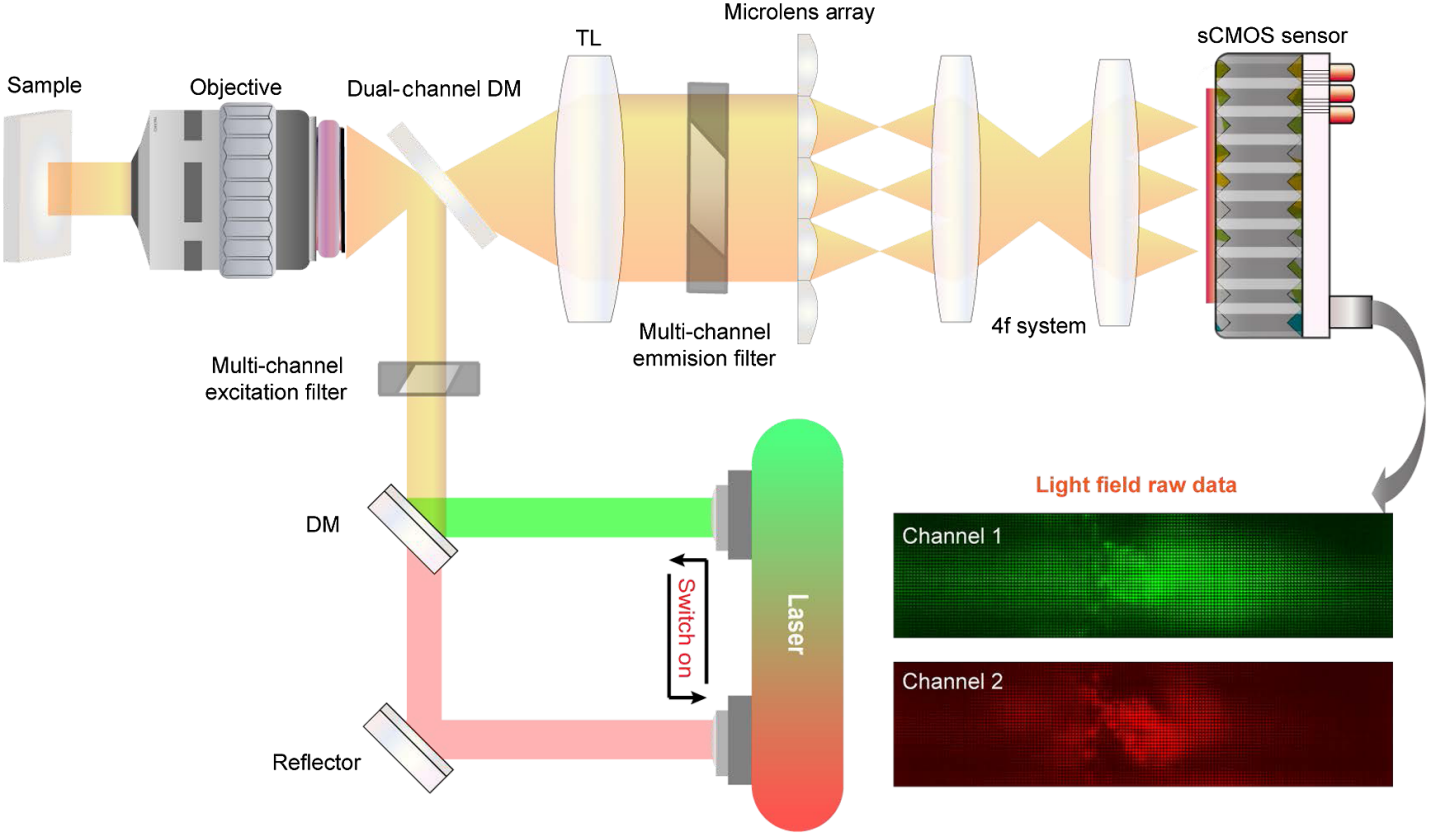
The schematic of the proposed time-division multiplexed double color light-field microscopy. The microlens array is placed at the conjugate plane of the sample. The sensor is aligned at the back focal plane of the microlens array to capture both the spatial and angular information within a snapshot, which is used for volumetric reconstruction of fluorescence samples using the proposed phase-space model in the Fourier domain. Laser sources at different wavelengths are utilized to implement the time-division spectral multiplexing for capturing different color channels of fluorescence samples at high-speed.

To further validate the effectiveness of the algorithm, we built up a multicolor LFM based on a commercially inverted microscope (Zeiss Observer Z1). As shown in Fig. 6, the sample was excited by multiple lasers, which can be switched on periodically at high speed. The switch of the laser is synchronized with the exposure of the camera to prevent color crosstalk. We used the dichroic mirror (DM) to combine two different laser beams. The dual-channel DM was employed to separate the excitation and emission light for multicolor epifluorescence imaging. We used a 20×/0.5 NA objective. Another 1.15× magnification was adopted to match the NA of the microlens array with a pitch size of 100  μm and F-number of 21. With an unfocused light field setup, to achieve maximum depth of range, we inserted the microlens array at the native image plane and used a 4f system to relay the back focal plane of the microlens array to the sensor plane, ensuring that every microlens covered around 13×13 sensor pixels. An sCMOS camera (Andor Zyla 4.2 plus) was used to collect the image of different fluorescence channels with a high sensitivity. The multicolor light field measurements were then processed sequentially and merged together after reconstruction with different pseudocolors.

**Fig. 7:**
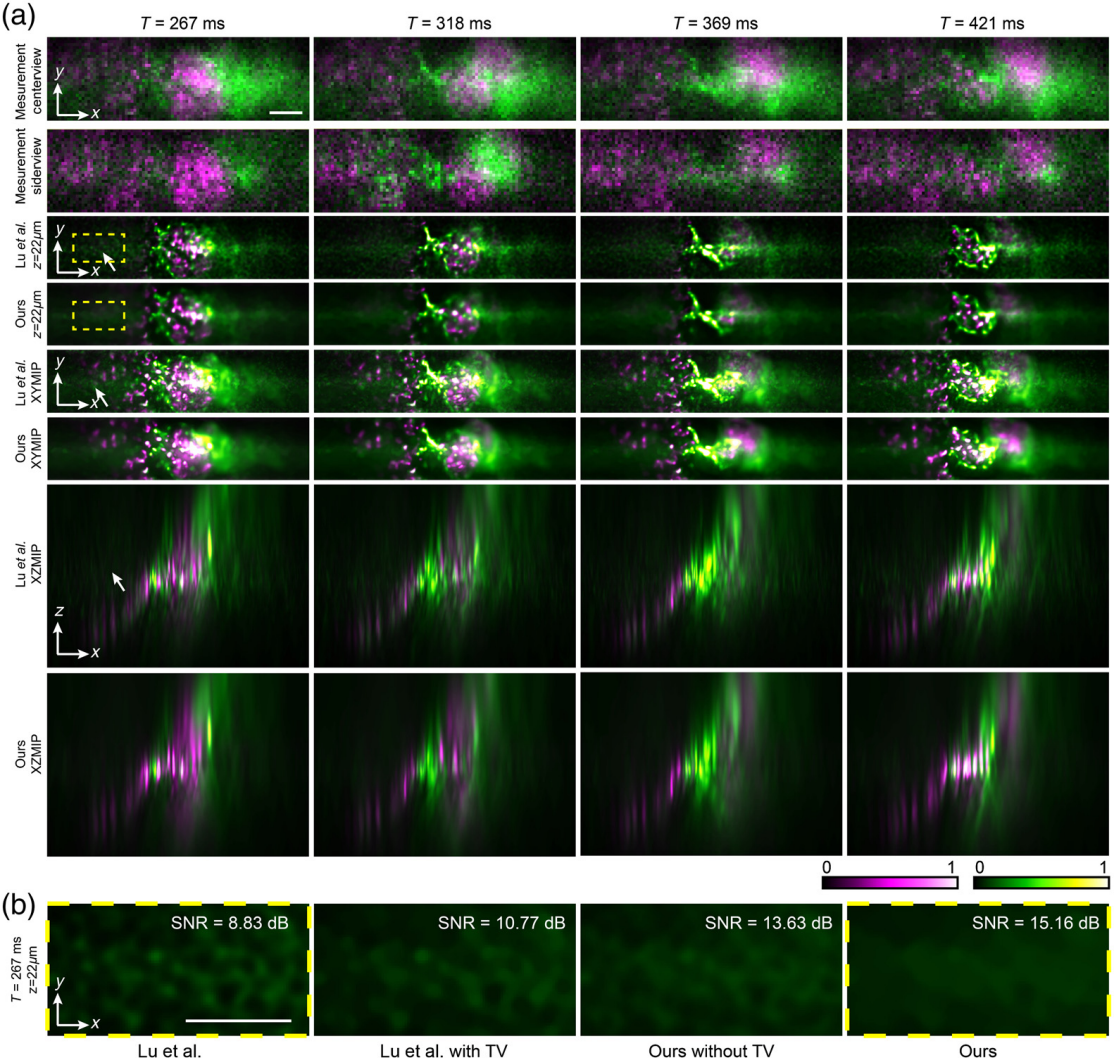
(a) Comparisons of the experimental data of the heart-beating process in a zebrafish larva. EGFP labels endothelial cells (green), and DsRed labels red blood cells (magenta). The video is captured at 97.5 Hz for each fluorescence channel. The results at different time stamps are shown in different columns with the whole video shown (Video 1, mp4, 45.2 MB [URL: https://doi.org/10.1117/1.JBO.27.7.076501.1]). The center view and a specific side view of the light field measurements (raw data of the system) are shown in the first two rows. The reconstruction results of a single slice and maximum intensity projections along different axes by different algorithms are shown separately in different rows. The white arrows indicate the artifacts introduced by the strong noise. (b) Ablation study on zebrafish larva experiment. Slice views and their region SNRs of four different reconstruction setups; the signal is defined as the mean of the pixel value in this area, and the noise is defined as the standard deviation. Our method showed better performance on the background area with low-light conditions. The yellow boxes indicate same background regions in (a). Scale bar, 50  μm.

To show the system performance, we imaged the 3D dynamics of the heart-beating process in a zebrafish larva at a high frame rate with a low light dose. The zebrafish larva used in this study had the ethical approval from the Animal Care and Use Committee of our affiliation. During imaging, the larva was embedded in 1% low-melting-point agarose in glass bottom dishes (D35-14-0-N, In Vitro Scientific). As shown in Fig. 7, we labeled both the endothelial cells by EGFP (green) and red blood cells by DsRed (magenta). The light-field video was captured at 195 Hz for two colors, corresponding to the 97.5-Hz frame rate for each color. To keep the high frame rate, the exposure time is only 2 ms for each frame, which leads to strong shot noise, which can be clearly observed from the center views and side views shown in Fig. 7(a). Such a huge noise reduces the contrast of the disparity of different views and results in the degradation of reconstruction performance. The traditional artifacts close to the native objective plane by previous methods^1^ were all successfully eliminated in the phase-space model. Compared with the previous ptychographic algorithm,^5^ the MLE results at different axial planes have much fewer background artifacts and speckle-like patterns, which severely reduce the image contrast, as indicated by the white arrows. These artifacts may be mistaken for the sample structures but can be clearly distinguished in the video. With TV regularizations, the noise can be further suppressed and structures can be even smoother, which can be observed after zooming in on the image. However, if we further increase the weight of TV regularization, the spatial resolution after reconstruction will be reduced. Such a tradeoff should be noticed during the use of the algorithm. With our 20× objective, the axial range that we achieve with similar resolution is around 100  μm, which is enough to cover the heart. The engine-like beating process of the heart can be clearly observed from both the images at different time stamps and in Video 1. The 3D motion of the blood cells can be readily tracked at such a high imaging speed. Such a 3D dynamic process could only be observed previously with very complicated and expensive imaging setups, whereas our multicolor LFM can do this in a much more compact and inexpensive way. Moreover, an ablation study with quantitative evaluation for our method is shown in Fig. 7(b); the zoomed-in slice view with a low-light region indicates that both random-subset ordering and TV regularization are helpful in reducing the shot noise in the reconstructed volume. However, as a wide-field imaging method, the background fluorescence in LFM still reduces the reconstruction performance and creates the artifacts for axial planes at the margin with larger intensity contributed by the background. Future work can remove this influence on quantitative fluorescence imaging.

## 4 Conclusion

We have derived the phase-space model of LFM in the Fourier domain based on wave optics. A Fourier phase-space deconvolution algorithm was then proposed for noise-robust 3D reconstructions in fluorescence imaging with low computational costs. By analyzing the reconstruction performance at different shot noise levels compared with the state-of-the-art method, we validated the improvement of computational effectiveness and noise robustness with random subset ordering. Further, we explored the performance of the sparse prior in fluorescence samples by applying the 3D total-variation regularization. We demonstrated a practical application of our algorithm in the observation of heart-beating dynamics in zebrafish larva with our custom-built multicolor LFM with low exposure time and low light dose.

A limitation of the current framework is that it is still an iterative algorithm with several iterations required for convergence. Although the ptychographic iteration of random subsets can accelerate the convergence process, the reconstruction process can be extended into a deterministic algorithm by an interpolation procedure in the Fourier domain as a generic Radon transform. More filtering processes can be incorporated into the Fourier domain at the same time.

For future work, other regularizations such as the Hessian constraint can be used to improve the noise robustness.^24^ Currently, only the spatial constraint has been considered in this work, and the time-lapse 3D video should have a strong sparsity in temporal domain. The low-rank constraints in time-lapse 3D video can also be added to further enhance the noise performance. As the deconvolution process can be modeled as a kind of tomography with axially-nonuniform PSF in the phase space, the same missing cone problem in computed tomography and magnetic resonance imaging exists in LFM, leading to a low resolution in axial dimension, which can be observed in our xz projections. Deep learning techniques can be applied to fill the gap in the Fourier domain with the prior of data structures, which have been verified in other tomographic algorithms.^25^^,^^26^

## Code, Data, and Materials Availability

The datasets generated and/or analyzed during the current study are available from the corresponding author on reasonable request.
